# Routine Vaccination During COVID-19: A Case of Maternal Neonatal Tetanus From Pakistan

**DOI:** 10.3389/frph.2021.790647

**Published:** 2023-01-12

**Authors:** Sehar Iqbal, Inayat Ali

**Affiliations:** ^1^College of Pharmacy, Al Ain University, Abu Dhabi, United Arab Emirates; ^2^Department of Public Health and Allied Sciences, Fatima Jinnah Women University, Rawalpindi, Pakistan; ^3^Department of Anthropology, Fatima Jinnah Women University, Rawalpindi, Pakistan; ^4^Department of Social and Cultural Anthropology, University of Vienna, Vienna, Austria

**Keywords:** COVID-19, routine immunization, tetanus vaccination, Pakistan, pandemic

## Introduction

Various factors—including natural disasters and health emergencies—considerably affect vaccination programs. The reasons can be multiple. This is visible during the 2 years of the ongoing coronavirus pandemic causing COVID-19, which has overwhelmed the entire world and yet fearing its consecutive waves of various variants, e.g., Omicron (1). With no exception, the significant health effects are associated with measures to contain COVID-19, such as physical distancing (2). Consequently, all routine immunization activities including Maternal Neonatal Tetanus (MNT) vaccination have been affected around the world, including in Pakistan (3).

## MNT: A Brief Overview

What is tetanus begs a brief overview. It is a life threatening disease caused by the toxin of gram-positive bacillus *Clostridium tetani* (*C. tetani*), which invades the body through any wound contamination (4). The neurotoxin of *C. tetani* affects the nervous system, spasms of respiratory, laryngeal, and abdominal muscles and ultimately results in a failure of respiration leading to death (5). Maternal tetanus toxoid (TT) vaccination is an important endeavor to prevent higher maternal mortalities caused by MNT infection (6). It is recommended since the 1980s and has been administered to millions of women for preventing maternal and neonatal tetanus (7).

However, MNT is still a substantial cause of significant maternal mortalities in low-income countries including Pakistan. Inadequate antenatal care (ANC) facilities, unsatisfactory vaccination coverage, unhygienic delivery practices are key causative factors for the prevalence of MNT in Pakistan (8, 9). WHO (4) recommended that women should initiate early ANC in the 1st trimester of conception for better feto-maternal outcomes. Yet, not a sufficient progress has been observed in Pakistan to seek ANC as the National Nutrition Survey data showed that a significant number of pregnant women (63.5%) sought ANC only during their last trimester of pregnancy (10).

Similarly, “disinfected” childbirth practices in homes and healthcare centers with provided sterilize instruments could also be an important preventive measure to reduce maternal neonatal tetanus toxoid (11). It has been reported that only 55% of births in Pakistan are attended by skilled health personnel (12). That means the remaining portion consults a Dai (traditional community midwife) who can be officially recognized and integrated frontline caregivers for decreasing the impact on the healthcare system of the country, and these should be given appropriate skills (13).

Furthermore, to minimize MNT, around 7 million pregnant women were immunized against vaccine-preventable diseases including TT vaccine in 2018 (14). Nonetheless, Pakistan is still one among the priority countries which have failed to achieve WHO's MNT elimination target of neonatal tetanus (< 1 per 1,000 live births per year) in all district of country (4). As mentioned earlier, this vaccination has further been affected by the pandemic.

## Discussion and Conclusion

The sustainable development goals 2030 emphasized the development of vaccines and medicines research with easy access to cheap essential medicines and vaccines specially for low-income countries (12). In addition, for preventing maternal and neonatal mortality, WHO has provided a global guideline for vaccination schedule to eliminate global MNT and to ensure lifetime protection against tetanus through tetanus-toxoid-containing vaccines such as tetanus-diphtheria (4) (see Table 1). However, there is evidence that when vaccination is halted, the vaccine-preventable diseases (VPDs) cause severe outbreaks (3), as it was observed during the 2013–16 Ebola outbreak in West Africa. The similar has also been observed during the ongoing pandemic. This mainly happened due to the interrupted vaccination programmes and shifting of resources toward the ongoing challenge.

**Table 1:** Immunization schedule of tetanus-diphtheria (Td) vaccines^a^.

**Tetanus-diphtheria (TD) schedule**	**1st dose**	**2nd dose**	**3rd dose**	**4th dose**	**5th dose**
Children >1 year of age, adolescents, adults and pregnant women^b^ (no previous immunization)	As early as possible	At least 4 weeks later after 1st dose	At least 6 months later after 2nd dose	At least 1 year later after 3rd dose	At least 1 year later after 4th dose
Pregnant women who received 3 childhood diphtheria tetanus pertussis (DTP) doses	As early as possible in first pregnancy	At least 4 weeks later after 1st dose, and 2 weeks before birth	At least 1 year later, or in next pregnancy		
Pregnant women who received 4 childhood DTP doses	As early as possible in first pregnancy	At least 1 year later, or in next pregnancy			
Supplementary immunization activities in high-risk areas, for women of reproductive age (WRA)	During round 1	During round 2, at least 4 weeks after round 1	During round 3, at least 6 months later, after round 2	At least 1 year later or in next pregnancy	At least 1 year later or in next pregnancy

^*a*^Developed from WHO global guidelines to obtain long-term protection against tetanus required to obtain long-term protection against tetanus.

^*b*^For pregnant women, the second dose should be administered at least 2 weeks before giving birth. Doses 3–5 may also be provided during subsequent pregnancies.

Similar to the entire vaccination program in Pakistan, the tetanus vaccination is significantly affected. During the COVID-19 pandemic, neither pregnant women might visit a healthcare facility to receive the required vaccine nor lady health workers could make it possible to visit these women to give them the required vaccine. It can be truly anticipated that many women will deliver their babies at homes not at any healthcare facilities (13). Consequently, there are greater chances that cases of tetanus would considerably increase during the ongoing pandemic.

Hence, it is highly necessary for the government to deal with the pressing context-specific issues that affect the vaccination programme in the country. Undoubtedly, all efforts should be made to run the routine vaccination drives during these overwhelming times to avert further challenges posed by other microorganisms.

## Author Contributions

SI and IA: conceptualization and writing—original draft, literature search, review, and validity. IA: critical reading, discussion, revisions, and editing. Both authors contributed to the article and approved the submitted version.

## References

[1] Ali I. COVID-19: are we ready for the second wave? Disaster Med Public Health Prep. (2020) 2020:1–3. 10.1017/dmp.2020.329 32379015 PMC7239772

[2] Ali I. Impact of COVID-19 on vaccination programs: adverse or positive? Hum. Vaccin Immunother. (2020) 16:1–7. 10.1080/21645515.2020.1787065 32961081 PMC7733893

[3] Ali I. Covid-19 and cancelled vaccination programs: forecasting outbreaks of vaccine preventable diseases (vpds) in pakistan. Vaccine X. (2022) 10:100151. 10.1016/j.jvacx.2022.100151 35229077 PMC8867976

[4] World Health Organization (WHO). Tetanus vaccines: WHO position paper, February 2017 – recommendations. Vaccine. (2018) 36:3573–5. 10.1016/j.vaccine.2017.02.034 28427847

[5] Hassel B. Tetanus: pathophysiology, treatment, and the possibility of using botulinum toxin against tetanus-induced rigidity and spasms. Toxins. (2013) 5:73–83. 10.3390/toxins5010073 23299659 PMC3564069

[6] Chu HY Englund JA. Maternal immunization. Clin Infect Dis. (2014) 59:560–8. 10.1093/cid/ciu327 24799324 PMC4168293

[7] Englund JA. Maternal immunization - promises and concerns. Vaccine. (2015) 33:6372–3. 10.1016/j.vaccine.2015.07.084 26263199

[8] Lambo JA Nagulesapillai T. Neonatal tetanus elimination in Pakistan: progress and challenges. Int J Infect Dis. (2012) 16:e833–42. 10.1016/j.ijid.2012.07.015 22940280

[9] Iqbal S Ali I Ekmekcioglu C Kundi M. Increasing frequency of antenatal care visits may improve tetanus toxoid vaccination coverage in pregnant women in Pakistan. Hum Vaccines Immunother. (2020) 16:1529–32. 10.1080/21645515.2019.1705693 32118509 PMC7482883

[10] Government of Pakistan UNICEF. National Nutrition Survey Pakistan 2011. Gov. Pakistan UNICEF Pakistan (2011). p. 1–104. Available online at: http://gilanifoundation.com/homepage/Free_Pub/HEALTH/National_Nutrition_Survey_of_Pakistan_2011.pdf (accessed August 23, 2020).

[11] Aqeel AY Arishi HM Ageel HI Arishi NH. Epidemiological and clinical aspects of neonatal tetanus from a tertiary care hospital. Int J Pediatr Adolesc Med. (2017) 4:71–4. 10.1016/j.ijpam.2016.10.001 30805505 PMC6372480

[12] World Health Organization. World Health Statistics 2017 : Monitoring Health for the SDGs. World Heal. Organ. (2017). p. 103. Available online at: https://www.who.int/gho/publications/world_health_statistics/2017/en/ (accessed August 23, 2020).

[13] Ali I Sadique S Ali S Davis-Floyd R. Birthing between the “Traditional” and the “Modern”: dāī practices and childbearing women's choices during COVID-19 in Pakistan. Front Sociol. (2021) 6:622223. 10.3389/fsoc.2021.622223 34026899 PMC8138426

[14] Government of Pakistan. Pakistan Economic Survey 2018-19. Econ. Advis. Wing, Financ. Div. Gov. Pakistan, Islam. (2019). p. 1–503. Available online at: http://www.finance.gov.pk/survey/chapters_19/Economic_Survey_2018_19.pdf (accessed August 23, 2020).

